# Sex-Specific Differences in the Associations Between Omega-6 Polyunsaturated Fatty Acids and Type 2 Diabetes in Chinese People

**DOI:** 10.3389/fnut.2021.739850

**Published:** 2021-10-22

**Authors:** Yingying Li, Hui Shen, Yike Li, Mei Bi, Yanhong Bi, Xiaoyu Che, Simiao Tian, Yazhuo Liu

**Affiliations:** ^1^Department of Clinical Nutrition and Metabolism, Affiliated Zhongshan Hospital of Dalian University, Dalian, China; ^2^Department of Research, Affiliated Zhongshan Hospital of Dalian University, Dalian, China

**Keywords:** omega-6 fatty acids, type 2 diabetes mellitus, sex-specific, arachidonic acid, n-6 PUFAs

## Abstract

**Background:** Some evidence indicates a potential beneficial effect of omega-6 polyunsaturated fatty acids (n-6 PUFAs) on type 2 diabetes mellitus (T2DM); however, the findings to date remains inconclusive and little is known about whether sex modifies these associations. Therefore, this study aimed to investigate potential sex-specific differences in this associations among Chinese adults.

**Methods:** We conducted a cross-sectional study in an area of Dalian city, China; Chinese men and women who attended the Department of Clinical Nutrition and Metabolism between January and December 2020 were invited to participate in this study. All participants were assessed for basic demographic characteristics, fasting blood glucose, HbA1c, and other serum biomarkers and serum phospholipid FAs.

**Results:** In total, 575 Chinese adult participants (270 men and 305 women) were included in the analysis. Hypertension and dyslipidaemia were more common among men than women, but there were no significant differences between the sexes in fatty acid composition, except for eicosadienoic acid (EA; 20:2n-6) and total monounsaturated fatty acids (MUFA). The age-adjusted OR for having T2DM in the highest quartile of arachidonic acid (20:4n-6) level was 0.47 (95% CI, 0.22, 0.98) in men, and this association remained consistently significant in the fully adjusted multivariate models. In contrast, no significant associations between n-6 PUFAs and T2DM risk were observed in women, regardless of model adjustment.

**Conclusions:** In conclusion, these results demonstrate a notable sex-specific differences in the associations between n-6 PUFAs and T2DM. Higher n-6 PUFA status may be protective against the risk of T2DM in men.

## Introduction

Diabetes has becoming one of the most important public health issues because of its increasing prevalence worldwide and its significant clinical complications (1). In 2014, an estimated 422 million people were diagnosed with diabetes globally (2), and that number is estimated to increase to 642 million by 2040 (3). Among the general Chinese adult population, the estimated number of adults with diabetes in 2007–2008 was 92.4 million (4) and this increased to 164 million in 2019 (5). Diabetes is a well-recognised risk factor for premature morbidity and mortality and affects all segments of the population (6). More importantly, it is estimated to be the seventh leading cause of death (7), and its related health complications are estimated to contribute to ~0.84 million deaths in China by 2030 (8). Therefore, appropriate treatment, lifestyle, and in particular, dietary strategies should be implemented to treat and prevent the onset of T2DM.

Among the various dietary factors, it has been suggested that n-6 PUFAs, particularly linoleic acid (18:2n-6) and arachidonic acid (20:4n-6), which can be found in plant and vegetable oils, may have potentially beneficial effect on T2DM and insulin sensitivity (9, 10); however, the findings related to n-6 biomarkers, metabolic dysfunction and health outcomes are mixed and inconclusive. Linoleic acid is the predominant n-6 PUFA and accounts for 80–90% of total dietary PUFAs (11), whereas arachidonic acid accounts for a minor component of total dietary PUFAs and can be obtained by means of dietary intake and the endogenous formation of linoleic acid (12). Early studies have suggested critical pro-inflammatory roles of linoleic acid and arachidonic acid (13); however, growing evidence indicates that n-6 and its main PUFA biomarkers are associated with a lower risk of T2DM, cardiometabolic diseases and their related biomarkers, including glycaemia and insulin resistance (14, 15). Recently, a large pooled analysis of 20 prospective cohort studies from 10 countries revealed that subjects in the highest quintile for the linoleic acid had a 35% lower risk of T2DM when compared with subjects the lowest quintile (16), and more importantly, the protective effect of linoleic acid was generally similar in phospholipids, plasma, cholesterol esters, and adipose tissue. However, another very extensive systematic review which included only randomised controlled trials (RCT) found little to no effect of n-6 supplementation on glucose metabolism and treatment of T2DM (9). Given these inconsistent findings, the association between n-6 PUFA biomarkers and T2DM remains inconclusive.

In addition, sex may serve as an important modifying variable in the relationship between n-6 PUFAs and T2DM risk because there are clear sex-specific disparities in dietary intake habits, which lead to varying n-6 PUFA profiles (17). To date, to the best of our knowledge, there are no studies investigating the potential link between T2DM and n-6 PUFAs in serum phospholipids in the Chinese population; there are also non-studies of whether the degree and direction of the relationships between n-6 PUFAs and T2DM differ by sex. Therefore, the aim of this study was to investigate potential sex differences in the associations between n-6 PUFAs and risk of T2DM among Chinese adults.

## Materials and Methods

### Study Population

This was a single centre observational study performed at the Affiliated Zhongshan Hospital of Dalian University, located in Dalian, China. The Chinese patients aged ≥18 years who attended the Department of Clinical Nutrition and Metabolism between January and December 2020 were invited to participate in the study. The study protocol was approved by the Institutional Ethics Committee of the Affiliated Zhongshan Hospital of Dalian University and conducted according to the Declaration of Helsinki. All methods were performed in accordance with the relevant guidelines and regulations. Written informed consent was obtained for analysis of demographics, medical histories and serum samples. Participants were invited if (1) they are Chinese people by ancestry, and lived in China for at least 5 years, (2) they were aged ≥18 years and (3) willing to participate in this survey. Of 719 participants invited, we excluded participants who were diagnosed with type 1 diabetes; those who were pregnant or lactating; those who reported a history of cardiovascular disease, cancer, mentally disabled, severe liver or kidney dysfunction; those who received lipid-lowering drugs in the long-term (such as statins and fibrates); and those who received a medically supervised diet program. Of the remaining 633 participants, we further excluded 58 participants who had no available measurement of either plasma glucose or glycated heamoglobin A1c (HbA_1C_), or had no complete data on serum phospholipid FA. Finally, 575 participants consented to and completed in the present study, and among them, main co-morbidities included metabolic abnormalities such as increased blood pressure (BP), impaired lipid profiles and reduced bone mineral density related to vitamin D deficiency.

### Data Collection

Participants' demographic information was collected by a standardised questionnaire which was completed by the participants during face-to-face interviews with well-trained personnel. The standardised questionnaire included information on (1) sociodemographic characteristics such as age, gender, marital status and education level; (2) behavioural factors such as smoking status, alcohol intake and physical activity; and (3) self-reported family history. Smoking status was defined as “Yes” (current or former) if the participant smoked at least one cigarette a day for more than 6 months; alcohol intake was defined as “Yes” (current or former) if the participant had a drink at least once a week on average.

Body weight and height were measured while the participants were barefoot and dressed in light clothing: weight and height was measured by digital weighing scale (BF-220, TANITA, Tokyo, Japan) and a wall-mounted stadiometer, and taken to the nearest 0.1 kg and 0.1 cm, respectively. Waist circumference was measured midway between the lowest rib and the iliac crest using a flexible anthropometric tape on the horizontal plane with the participant in the standing position; waist circumference was measured to the nearest 0.1 cm. Body mass index (BMI) was calculated by dividing the participant's weight in kilogrammes by the square of their height in metres. Systolic and diastolic BPs (SBP and DBP) were measured on the right arm using a validated semiautomatic oscillometer (OMRON Model1 Plus; Omron Company, Kyoto, Japan). Measurements were collected in triplicate after seated rest for 10 min; the mean of the three measurements was used in the analyses.

Fasting blood samples were collected in the morning following an overnight fast. Plasma and serum samples were then frozen and stored at −80°C for later laboratory analysis. Serum level of fasting plasma glucose (FPG) was measured using the hexokinase glucose-6-phosphate dehydrogenase method (ADVIA1650 Chemistry System, Bayer, Leverkusen, Germany), and the fasting serum lipid profiles such as total cholesterol (TC), high-density lipoprotein cholesterol (HDL-C) concentration, triglycerides (TG) were measured by enzymatic colorimetric method (HITACHI-7170, Hitachi, Tokyo, Japan). In addition, HbA_1c_ was determined by the method established by the high-performance liquid chromatography (HPLC) method (BIO-RAD, Hercules, CA, USA). All blood samples were measured at a central, certified laboratory in Dalian with strict quality control.

### Measurement of FA Composition

Serum FAs were measured from samples stored at −80°C using gas chromatography by means of an AB SCIEX Triple Quad 4500MD liquid chromatography-mass spectrometery (LCMS-4500MD) held at the Institute of Metal Research Chinese Academy of Sciences (located at Dalian city). A detailed description of the laboratory analyses has been provided previously (18). In brief, serum FAs were extracted from whole blood using the Folch et al. method (19) with chloroform-methanol (2:1). The chloroform phase was evaporated and treated with sodium methoxide, which methylated the esterified fatty acids. The oven temperature increased by 5°C/min from 140°C to 220°C during the analysis run. The peaks were identified on the basis of retention times recorded for different standards. FA levels were quantified by gas-liquid chromatography with reference standards purchased from NU-Cheque Prep, Inc, and each analyte had an individual reference standard. The FAs were expressed as as absolute concentrations in serum (nmol/ml) based on the quantity of eicosane used as an internal standard. The intra- and inter-assay coefficients of variation (CV) % for individual FAs, which were calculated using two serum samples as quality controls added to each batch, were ≤ 10 and ≤ 12%, respectively.

### Ascertainment of Outcome Variable T2DM

Participants were defined as having T2DM if their HbA_1C_ levels exceeded 6.5% or if they were currently under medical treatment for T2DM according to the American Diabetes Association criteria for the diagnosis of diabetes, regardless of fasting glucose levels (20).

### Definition of Exposure Variables

In this study, patients were diagnosed with hypertension if they had a systolic blood pressure of ≥140 mmHg and/or a diastolic blood pressure of ≥90 mmHg or if they self-reported use of antihypertensive medication (21). Patients were diagnosed with dyslipidemia were diagnosed if they fulfilled the following criteria: LDL-cholesterol ≥4.14 mmol/L, HDL-cholesterol <1.036 mmol/L and triglycerides ≥2.26 mmol/L (22).

### Statistical Analyses

The characteristics of the study population were presented as the mean and SD or median (interquartile range; IQR) for continuous variables, where appropriate, and as numbers and percentages for categorical variables. Comparisons between two sex groups were assessed by using Student's *t*-test or the Mann Whitney-*U*-test for continuous variables, and chi-square tests for categorical variables. In the examination of bivariate relationships between serum phospholipid n-6 PUFAs and T2DM biomarkers (e.g., FPG and HbA1C), the analysis was performed separately by sex and Spearman partial correlations were used, adjusting for age and BMI. Multivariate logistic regression analysis was conducted to estimate the associations between T2DM and both total and individual n-6 PUFAs, as well as other FAs, with odds ratios (ORs) and 95% confidence intervals (CIs) calculated. This analysis was also performed separately by sex and several different models with sequential adjustments for key potential confounders were fitted. Model 1 was adjusted for age; Model 2 was additionally adjusted for smoking status, alcohol status and BMI (model 2); and Model 3 was further adjusted for hypertension and dyslipidaemia. All statistical analyses were performed using R version 3.6.3 software (23). A *P*-value <0.05 was considered statistically significant.

## Results

In this study, a final sample included 575 adult participants (270 men and 305 women) with a mean age of 58.23 ± 11.94 years. There were significant differences between men and women in several anthropometric and biochemical variables (Table 1). Men had significantly higher BP, FPG, TG and UA levels compared to women, whereas they had lower TC, HDL-C and LDL-C levels than women. With regard to circulating serum fatty acid composition, no significant differences were observed between men and women among all except EA and total MUFA. Moreover, smoking and alcohol intake were more prevalent among men than women (smoking: 44.4 vs. 1.31%; alcohol intake: 40 vs. 4.92%, respectively). Hypertension and dyslipidaemia were more common among men than women, with both prevalent among more 40% of men and nearly 30% of women, respectively.

**Table 1:** Basic characteristics of studied sample.

	**Total sample (*n* = 575)**	**Men (*n* = 270)**	**Women (*n* = 305)**	***P*-value**
Age, years	58.23 ± 11.94	58.06 ± 12.31	58.38 ± 11.62	0.748
Smoker, *n* (%)	124 (21.57%)	120 (44.44%)	4 (1.31%)	<0.001
Alcohol drinker, *n* (%)	123 (21.39%)	108 (40.00%)	15 (4.92%)	<0.001
Weight, kg	70.47 ± 12.85	77.88 ± 11.78	63.90 ± 9.84	<0.001
Height, cm	168.32 ± 7.68	174.19 ± 5.49	163.12 ± 5.18	<0.001
BMI, kg/m^2^	24.76 ± 3.43	25.62 ± 3.35	23.99 ± 3.33	<0.001
SBP, mm Hg	130.91 ± 19.18	133.93 ± 19.61	128.24 ± 18.41	<0.001
DBP, mm Hg	81.78 ± 10.61	84.07 ± 10.61	79.76 ± 10.22	<0.001
FPG, mmol/L	6.01 ± 2.57	6.15 ± 2.30	5.89 ± 2.79	0.219
HbA_1C_, %	6.27 ± 1.48	6.38 ± 1.54	6.16 ± 1.43	0.081
TC, mmol/L	5.18 ± 1.09	4.97 ± 1.04	5.37 ± 1.11	<0.001
HDL-C, mmol/L	1.44 ± 0.45	1.33 ± 0.49	1.55 ± 0.38	<0.001
LDL-C, mmol/L	3.52 ± 13.75	2.82 ± 0.78	4.14 ± 18.85	0.223
TG, mmol/L	1.82 ± 1.27	2.06 ± 1.56	1.61 ± 0.89	<0.001
UA, μmol/L	337.06 ± 99.75	379.79 ± 98.44	299.38 ± 84.69	<0.001
Hypertension, *n* (%)	224 (38.96%)	124 (45.93%)	100 (32.79%)	0.002
Dyslipidaemia, *n* (%)	217 (37.74%)	115 (42.59%)	102 (33.44%)	0.03
Total SFAs	5,961.08 ± 2,567.60	6,125.20 ± 2789.68	5,815.79 ± 2,348.70	0.154
Total MUFAs	2,767.82 ± 1,622.26	2,991.01 ± 1917.91	2,570.24 ± 1,277.03	0.002
Total PUFAs	7,466.41 ± 2,420.70	7,391.04 ± 2584.75	7,533.12 ± 2,267.82	0.486
Total n-3 PUFAs	711.61 ± 369.14	705.54 ± 390.65	716.98 ± 349.56	0.713
Total n-6 PUFAs	6,431.79 ± 2,054.92	6,358.43 ± 2177.27	6,496.72 ± 1,941.49	0.424
Linoleic acid (18:2n-6)	5,227.26 ± 1,654.26	5,182.05 ± 1784.64	5,267.29 ± 1,531.48	0.542
Eicosadienoic acid (20:2n-6)	31.99 ± 15.57	33.66 ± 17.49	30.51 ± 13.49	0.017
Arachidonic acid (20:4n-6)	1,204.52 ± 552.03	1176.38 ± 533.40	1229.44 ± 567.71	0.249

*Data are reported as the mean (SD) or n (%). Comparison of two sex groups was conducted by Student t-test for continuous variables or χ2 tests for categorical variables. BMI, body mass index; SBP, systolic blood pressure; DBP, diastolic blood pressure; FPG, fasting plasma glucose; HbA_1c_, heamoglobin A1c; TC, total cholesterol; HDL-C, high-density lipoprotein cholesterol; LDL-C, low-density lipoprotein cholesterol; TG, triglycerides; UA, uric acid; SFA, saturated fatty acid; MUFA, monounsaturated fatty acids; PUFA, polyunsaturated fatty acid*.

The overall and sex-specific distributions of serum FA compositions between participants with and without diabetes are shown in Table 2. Among overall population, none of these FA compositions showed any significant differences between two diabetic status; however, participants with T2DM had lower levels of total n-3 PUFAs, n-6 PUFAs, and PUFAs, and arachidonic acid compared with those without T2DM, in contrast a slight higher levels of linoleic acid, EA, total saturated fatty acids (SFAs) and MUFAs was found in participants with diabetes. With regard to men, a consistent lower levels of serum FA compositions was noted in men with diabetes when compared to their peers without, whereas for women, a contrast pattern was found such that women with diabetes had a slight higher levels of each FA composition than women without diabetes.

**Table 2:** Distribution of serum fatty acids according to diabetic status among Chinese men and women.

	**Total sample (*****n*** **=** **575)**			**Men (*****n*** **=** **270)**			**Women (** ***n*** **=305)**		
	**Without T2DM** **(*n* = 406)**	**With T2DM** **(*n* = 169)**	***P*-value**	**Without T2DM** **(*n* = 180)**	**With T2DM** **(*n* = 90)**	***P*-value**	**Without T2DM** **(*n*=226)**	**With T2DM** **(*n*=79)**	**P value**
Total SFAs	5, 949.65, 2, 523.59	5, 988.53, , 677.85	0.872	6, 288.67, 2, 862.75	5, 798.25, 2, 622.40	0.162	5679.63, 2185.94	6205.31, 2740.22	0.126
Total MUFAs	2, 739.73, 1, 624.78	2, 835.32, 1, 619.00	0.52	31793015.78, 11793885.77	2, 941.49, 1, 990.43	0.769	2519.86, 1346.55	2714.36, 1047.47	0.191
Total PUFAs	7, 521.15, 21793398.17	7, 334.89, 2, 476.26	0.408	7, 530.09, 2, 555.17	7, 112.96, 2, 635.23	0.217	7514.03, 2271.11	7587.71, 2271.95	0.804
Total n-3 PUFAs	721.64, 368.60	687.50, 370.42	0.314	729.04, 381.76	658.54, 405.92	0.172	715.75, 358.51	720.50, 324.73	0.914
Total n-6 PUFAs	6, 476.27, 2, 036.90	61793324.91, 21793099.84	0.428	6, 472.76, 21793164.31	6, 129.77, 21793197.19	0.226	6479.07, 1934.31	6547.23, 1973.45	0.791
Linoleic acid (18:2n-6)	5, 254.06, 1, 639.96	5, 162.89, 1, 691.30	0.553	5, 252.41, 1, 781.02	5, 041.34, 1793.50	0.362	5255.38, 1522.34	5301.36, 1566.64	0.821
Eicosadienoic acid (20:2n-6)	32.08, 15.63	31.77, 15.46	0.828	33.79, 16.96	33.41, 18.61	0.871	30.72, 14.38	29.91, 10.61	0.596
Arachidonic acid (20:4n-6)	1, 222.21, 539.16	1, 162.02, 581.22	0.249	1, 220.35, 524.50	1, 088.43, 543.07	0.059	1223.69, 551.71	1245.87, 614.59	0.777

*T2DM, type 2 diabetes mellitus; SFA, saturated fatty acid; MUFA, monounsaturated fatty acids; PUFA, polyunsaturated fatty acid*.

Spearman partial correlations between each serum FA biomarker and FPG and HbA_1C_ were calculated after adjusting for age and BMI, in both the overall sample and sex-specific samples (Table 3). Among the total sample, there were weak but significant positive correlations between HbA_1C_ and all except linoleic acid and EA; however, these correlations became non-significant inverse for total n-6 PUFAs, linoleic acid, arachidonic acid and EA with FPG. In men, there were inverse and non-significant correlations between these FAs and FPG, and these correlations became positive for HbA_1C_. In women, all FAs except EA were positively correlated with both FPG and HbA_1C_, but the significant positive correlations were only observed between HbA_1C_ biomarker and FAs except total n-6 PUFAs, linoleic acid and EA in women.

**Table 3:** Spearman partial correlations between serum fatty acid biomarkers and FPG and HbA_1C_ stratified by sex.

		**FPG**	**HbA_**1c**_**
		**Partial-r**	**Partial-r**
Total sample	Total SFAs	0.016	0.156*
	Total MUFAs	0.052	0.117*
	Total PUFAs	−0.024	0.091*
	Total n-3 PUFAs	0.024	0.131*
	Total n-6 PUFAs	−0.030	0.082*
	Linoleic acid (18:2n-6)	−0.027	0.070
	Eicosadienoic acid (20:2n-6)	−0.028	0.038
	Arachidonic acid (20:4n-6)	−0.038	0.104*
Men	Total SFAs	−0.072	0.107
	Total MUFAs	0.008	0.069
	Total PUFAs	−0.046	0.098
	Total n-3 PUFAs	−0.001	0.106
	Total n-6 PUFAs	−0.052	0.097
	Linoleic acid (18:2n-6)	−0.047	0.092
	Eicosadienoic acid (20:2n-6)	−0.008	0.034
	Arachidonic acid (20:4n-6)	−0.081	0.067
Women	Total SFAs	0.121*	0.214*
	Total MUFAs	0.094	0.1771*
	Total PUFAs	0.018	0.098
	Total n-3 PUFAs	0.070	0.174*
	Total n-6 PUFAs	0.013	0.083
	Linoleic acid (18:2n-6)	0.011	0.057
	Eicosadienoic acid (20:2n-6)	−0.057	0.045
	Arachidonic acid (20:4n-6)	0.018	0.158*

*Correlations adjusted for age and BMI.*

*P < 0.05. FPG, fasting plasma glucose; HbA_1c_, heamoglobin A1c; SFA, saturated fatty acid; MUFA, monounsaturated fatty acids; PUFA, polyunsaturated fatty acid.

The sex-specific associations between FA biomarkers and T2DM are presented in Table 4. In men, among the n-6 PUFAs, arachidonic acid was significantly and inversely associated with T2DM, with age-adjusted OR value of 0.47 (95% CI, 0.22, 0.98) when comparing the highest quartile of arachidonic acid to the lowest (Model 1). This association was consistently significant in two fully adjusted multivariate models (Model 2 and Model 3). The ORs (95% CIs) of T2DM in the model 3 for the lowest through the highest quartiles of arachidonic acid were 1.00, 0.62 (0.3, 1.28), 0.59 (0.28, 1.24), and 0.42 (0.19, 0.92), respectively. With regard to other n-6 PUFAs, higher level of linoleic acid and eicosadienoic acid were likely to reduce the chance of having T2DM compared to lower levels (i.e., lowest quartile), but none of these associations were statistically significant either in the basic age-adjusted model or the fully multivariable-adjusted model. Furthermore, none of total n-3 PUFAs, SFAs nor MUFAs were significantly associated with T2DM, regardless of whether the model was adjusted.

**Table 4:** Association between serum FAs and risk of type 2 diabetes mellitus stratified by Chinese men and women.

	**Model**	**Men**				**Women**			
		**1st Quartile**	**2nd Quartile**	**3rd Quartile**	**4th Quartile**	**1st Quartile**	**2nd Quartile**	**3rd Quartile**	**4th Quartile**
Total SFAs	Model 1	1 (reference)	0.71 (0.35, 1.46)	1.09 (0.51, 2.33)	0.58 (0.28, 1.22)	1 (reference)	0.77 (0.33, 1.77)	1.35 (0.66, 2.76)	1.62 (0.76, 3.44)
	Model 2	1 (reference)	0.70 (0.34, 1.45)	1.00 (0.46, 2.17)	0.53 (0.25, 1.12)	1 (reference)	0.71 (0.30, 1.68)	1.28 (0.61, 2.69)	1.42 (0.65, 3.10)
	Model 3	1 (reference)	0.70 (0.33, 1.46)	0.93 (0.42, 2.05)	0.49 (0.22, 1.10)	1 (reference)	0.74 (0.31, 1.78)	1.26 (0.59, 2.71)	1.49 (0.63, 3.52)
Total MUFAs	Model 1	1 (reference)	0.67 (0.32, 1.43)	0.86 (0.41, 1.80)	0.67 (0.32, 1.42)	1 (reference)	0.61 (0.28, 1.34)	0.79 (0.37, 1.67)	1.57 (0.76, 3.25)
	Model 2	1 (reference)	0.64 (0.30, 1.37)	0.78 (0.37, 1.65)	0.58 (0.27, 1.26)	1 (reference)	0.58 (0.25, 1.31)	0.68 (0.31, 1.47)	1.39 (0.65, 2.95)
	Model 3	1 (reference)	0.64 (0.30, 1.39)	0.73 (0.33, 1.58)	0.48 (0.20, 1.19)	1 (reference)	0.56 (0.24, 1.28)	0.68 (0.30, 1.52)	1.54 (0.64, 3.72)
Total PUFAs	Model 1	1 (reference)	0.87 (0.43, 1.75)	0.67 (0.32, 1.42)	0.76 (0.36, 1.59)	1 (reference)	1.04 (0.47, 2.30)	1.08 (0.50, 2.33)	1.22 (0.57, 2.61)
	Model 2	1 (reference)	0.86 (0.42, 1.74)	0.60 (0.28, 1.30)	0.73 (0.35, 1.56)	1 (reference)	1.14 (0.50, 2.61)	1.05 (0.47, 2.32)	1.26 (0.57, 2.77)
	Model 3	1 (reference)	0.85 (0.41, 1.73)	0.57 (0.26, 1.26)	0.66 (0.30, 1.49)	1 (reference)	1.08 (0.47, 2.50)	0.97 (0.42, 2.24)	1.19 (0.51, 2.76)
Total n-3 PUFAs	Model 1	1 (reference)	1.04 (0.51, 2.13)	0.42 (0.19, 0.92)	0.85 (0.41, 1.75)	1 (reference)	0.83 (0.38, 1.83)	1.07 (0.50, 2.29)	1.11 (0.52, 2.37)
	Model 2	1 (reference)	0.93 (0.45, 1.93)	0.39 (0.17, 0.87)	0.79 (0.38, 1.64)	1 (reference)	0.85 (0.37, 1.92)	0.95 (0.43, 2.10)	1.08 (0.49, 2.38)
	Model 3	1 (reference)	0.92 (0.44, 1.91)	0.40 (0.18, 0.89)	0.75 (0.35, 1.61)	1 (reference)	0.84 (0.37, 1.91)	0.94 (0.42, 2.13)	1.05 (0.45, 2.45)
Total n-6 PUFAs	Model 1	1 (reference)	0.85 (0.42, 1.73)	0.99 (0.49, 2.00)	0.69 (0.32, 1.49)	1 (reference)	1.16 (0.52, 2.57)	1.22 (0.56, 2.68)	1.31 (0.61, 2.82)
	Model 2	1 (reference)	0.81 (0.39, 1.67)	0.90 (0.44, 1.85)	0.69 (0.31, 1.50)	1 (reference)	1.12 (0.49, 2.55)	1.13 (0.50, 2.53)	1.30 (0.59, 2.85)
	Model 3	1 (reference)	0.82 (0.40, 1.71)	0.85 (0.41, 1.79)	0.64 (0.28, 1.46)	1 (reference)	1.07 (0.47, 2.45)	1.06 (0.45, 2.49)	1.23 (0.54, 2.83)
Linoleic acid (18:2n-6)	Model 1	1 (reference)	0.98 (0.48, 2.00)	1.10 (0.54, 2.22)	0.83 (0.39, 1.78)	1 (reference)	0.79 (0.36, 1.77)	1.26 (0.59, 2.69)	0.99 (0.46, 2.10)
	Model 2	1 (reference)	0.94 (0.46, 1.95)	1.00 (0.48, 2.05)	0.82 (0.38, 1.78)	1 (reference)	0.77 (0.34, 1.75)	1.24 (0.57, 2.69)	0.95 (0.43, 2.06)
	Model 3	1 (reference)	0.92 (0.44, 1.91)	0.93 (0.44, 1.96)	0.76 (0.34, 1.74)	1 (reference)	0.72 (0.31, 1.66)	1.20 (0.54, 2.68)	0.92 (0.40, 2.09)
Eicosadienoic acid (20:2n-6)	Model 1	1 (reference)	1.35 (0.62, 2.92)	1.60 (0.75, 3.38)	1.12 (0.52, 2.40)	1 (reference)	0.84 (0.40, 1.75)	0.83 (0.40, 1.71)	0.75 (0.35, 1.60)
	Model 2	1 (reference)	1.24 (0.56, 2.74)	1.57 (0.73, 3.35)	1.02 (0.46, 2.24)	1 (reference)	0.84 (0.39, 1.79)	0.86 (0.41, 1.82)	0.68 (0.31, 1.52)
	Model 3	1 (reference)	1.20 (0.54, 2.67)	1.57 (0.71, 3.43)	0.96 (0.41, 2.25)	1 (reference)	0.78 (0.36, 1.68)	0.77 (0.35, 1.68)	0.64 (0.27, 1.50)
Arachidonic acid (20:4n-6)	Model 1	1 (reference)	0.63 (0.31, 1.28)	0.59 (0.29, 1.23)	**0.47 (0.22, 0.98)**	1 (reference)	1.10 (0.50, 2.43)	1.37 (0.64, 2.92)	0.93 (0.42, 2.08)
	Model 2	1 (reference)	0.62 (0.30, 1.26)	0.59 (0.28, 1.24)	**0.44 (0.21, 0.94)**	1 (reference)	1.15 (0.51, 2.61)	1.35 (0.62, 2.96)	0.97 (0.42, 2.20)
	Model 3	1 (reference)	0.62 (0.30, 1.28)	0.59 (0.28, 1.24)	**0.42 (0.19, 0.92)**	1 (reference)	1.07 (0.47, 2.46)	1.27 (0.58, 2.82)	0.92 (0.40, 2.14)

*Values are ORs (95% CIs) by quartile unless otherwise indicated. SFA, saturated fatty acid; MUFA, monounsaturated fatty acids; PUFA, polyunsaturated fatty acid. Model 1: adjusted for age; Model 2: Adjusted for Model 1 + smoking status, alcohol status and BMI; Model 3: Adjusted for Model 2 + hypertension and dyslipidaemia. The bold values mean the results are significant*.

In women, neither of total n-6 PUFAs nor individual n-6 PUFAs were found significantly associated with T2DM, although there were inverse association patterns for linoleic acid, arachidonic acid and EA. However, compared to the lowest quartile, having T2DM was higher in the highest quartile of both SFAs and MUFAs, but these positive associations were not statistically significant; moreover, a flat J-shaped relationships for both SFAs and MUFAs seemed to be found, with a decrease in OR of T2DM in the second quartile, after which ORs moderately rose to 1.49 and 1.54, respectively, but remaining no significant association.

## Discussion

In the present study, the serum concentration of arachidonic acid was inversely associated with T2DM in Chinese men but not in Chinese women, indicating a sex-specific effect in this association. There were inverse association patterns between total n-6 PUFAs, linoleic acid and T2DM; however, these associations were non-significant after adjustment for multiple confounders, regardless of sex. To the best of our knowledge, this is the first study to investigate sex-dependent associations between n-6 PUFAs and T2DM in the Chinese population.

The current finding of a reduced change of having T2DM with a higher concentration of serum arachidonic acid is consistent with several studies in Western countries. In the KIHD (Kuopio Ischaemic Heart Disease) case-cohort study, Yary et al. reported significant inverse associations between the risk of incident T2DM and both serum arachidonic acid and the D5D, a measure of arachidonic acid-based desaturase activity, in a 19-y follow-up study (15). In another prospective cohort study comprising 407 overweight, middle-aged men and women, Takkunen et al. found a very similar result that both serum arachidonic acid and D5D were significantly and inversely associated with the risk of T2DM, and for every one-unit increase in serum arachidonic acid, there was a 20% lower incidence of T2DM during the median follow-up of 11 years (18). The present findings are also consistent with several Asian studies and meta-analyses, although most did not find significant associations. For example, in a prospective study of Japanese employees, the incidence of T2DM exhibited a non-significant decreasing trend with elevated circulating serum arachidonic acid (24). Consistently, in a recent meta-analysis which pooled 39,740 adults from 20 prospective cohort studies (16), higher arachidonic acid was inversely associated with T2DM risk, but this was only statistically significant in plasma; the authors concluded that independent of lipid compartments, higher arachidonic acid does not contribute to T2DM development.

Arachidonic acid is a proinflammatory marker of glucose metabolism and weight regulation (25); thus, higher arachidonic acid may lead to an increased risk of T2DM from a theorectical perspective. However, the current study and several abovementioned studies suggested an inverse association with T2DM, and several mechanisms behind these associations have been suggested. For instance, arachidonic acid is a precursor to several anti-inflammatory metabolites (26) and a systematic review provided solid evidence indicating that higher concentrations of arachidonic acid are associated with a lower risk of coronary heart disease incidence (27). Therefore, the association between arachidonic acid and disease risk is difficult to predict solely on the basis of its effects on proinflammatory factors. Moreover, previous experimental studies have reported a protective effect of arachidonic acid on pancreatic b cells in a chemical-induced cytotoxicity model; this effect was related to increased antioxidant status and suppression of cytokine production (28, 29). This above mechanism emphasises that arachidonic acid could preserve insulin production capacity, meaning that the inverse association observed in this study is biologically plausible.

Several prospective and observational studies have reported relationships between PUFAs and risk of T2DM, but few studies have focused on sex-specific differences in these associations. Abbott et al. were among the first to address the sex differences in the associations between PUFA biomarkers and T2DM among the Western population (30). Further, in the RHLS (Retirement Health and Lifestyle Study) (31) and a meta-analysis of RCTs (32), the authors reported significant inverse relationships between n-3 PUFAs and both insulin resistance and T2DM in women but not men, indicating a clear sex-specific difference and suggesting a protective effect of n-3 PUFAs on insulin resistance and T2DM in the female population. However, in a prospective cohort comprising only 2189 middle-aged and older Finnish men, Yary et al. found that serum n-6 PUFAs, including linoleic acid and arachidonic acid, were inversely associated with the risk of incident T2DM (15); compared to the lowest quartile, men in the highest serum linoleic acid and arachidonic acid quartiles had a 48 and 38% lower hazard ratio of incident T2DM, respectively (15). These findings are consistent with the current study where that arachidonic acid was inversely associated with T2DM in men but not in women. The mechanisms by which n-6 PUFAs reduce the chance of having T2DM in men but not in women are not fully understood, but several have been suggested, including sex differences in n-6 PUFA distribution (33), exogenous dietary intake (34), and sex hormone-mediated adipose tissue distribution (35, 36). Further research is required to investigate the mechanisms underlying the sex-dependent association observed in this study.

Several prospective, observational and meta-analysis studies among Western and Asian populations have investigated the relationships between concentrations of total n-6 PUFAs, linoleic acid and T2DM risk, but the findings are conflicting; some have reported significant inverse associations (14, 16, 18, 37), whereas others have not (24, 38). In the EPIC (European Prospective Investigation into Cancer and Nutrition)-InterAct study, Forouhi et al. provided robust evidence of strong and significant inverse associations between T2DM and both total circulating plasma n-6 PUFAs and linoleic acid; the risk decreased by 13 and 20% for every 1-SD increase, respectively (14). Consistently, in the above-mentioned recent meta-analysis (16) and another prospective study comprising three large American cohorts (39), higher concentrations of total blood n-6 PUFAs and linoleic acid were associated with a lower risk of T2DM, independent of lipid compartment. However, the current study observed non-significant inverse relationships between T2DM and both total n-6 PUFAs and linoleic acid. In line with the current findings, several other studies have found similar but non-significant association patterns between T2DM and total n-6 PUFAs (24) and linoleic acid (24, 40) after adjustment for confounders, especially BMI. For instance, in a prospective study in the Japanese population, higher concentrations of total n-6 PUFAs and linoleic acid were found to be significantly associated a lower risk of T2DM before BMI adjustment, but these associations were moderately attenuated and became non-significant when adjusting for BMI. Similarly, in another multi-ethnic atherosclerosis study, although a significant inverse association was observed between linoleic acid and T2DM risk among the total sample, the association was non-significant among the subgroup of Chinese people (37). It can be speculated that the reasons for these differences may be due to differences in major dietary sources of n-6 PUFAs and linoleic acid. Considering the potential weight-reducing effect of LA (41), it may have long-term benefits in preventing T2DM risk, at least in part, by means of its effect on body fat.

In addition to studying the risks of endogenous PUFAs on T2DM, exogenous supplementary PUFAs are often used clinically. Many studies have investigated the role of intake of various FAs in T2DM prevention; for instance, higher intake of PUFA (42), n-6 FAs (9, 39) and n-3 FAs (42), as well as lower intake of saturated FAs (43) and trans-fatty acids (42), is recommended. However, the role of endogenous intake of PUFAs remains controversial. Of note, few prospective and RCT studies have focused on n-6 intake for T2DM prevention (39, 44–48), and the majority observed a null or very week associations between T2DM and n-6 FA intake, with relative risk values ranging from 0.97 to 1.01. In a very recent dose-response meta-analysis, Neuenschwander et al. reported null findings with regard to the associations between T2DM and intake of total n-6 and linoleic acid (49). This finding is inconsistent with another recent meta-analysis of RCTs (9), in which the authors suggested a favourable effect of higher n-6 FA intake in T2DM prevention. These discrepancies, as Neuenschwander et al. argue, may be due to the very limited number of included trials (only 2 trials) and the variation in the control groups from Brown et al. meta-analysis study, which could attenuate the strength of the evidence. Together with the findings from previous prospective and interventional studies of T2DM, the current findings do not suggest that high levels of n-6 PUFAs are harmful; however, given the inconsistent data, more research is warranted to clarify the associations between n-6 PUFAs, linoleic acid and T2DM.

The notable strengths of this study should be mentioned. First, assessment for diabetes was based on objective measurements including HbA_1c_ and blood glucose as well as self-report data. Second, FA metabolism profile was determined using liquid-phase tandem mass spectrometry, which guarantees the rigorous quality of the data. Nonetheless, this study also has several limitations that should be acknowledged. First, due to the nature of the cross-sectional design, this study could only examine the associations and related magnitude between n-6 PUFAs and T2DM and the causality could not be determined. Cohort studies are warranted to clarify the current findings. Second, despite study sample of inpatients, the participants with unstable clinical situations or severe diseases were excluded to minimise the sample bias. Meanwhile, the analyses were adjusted for multiple potential confounders and co-morbidities to minimise another possibility of bias. Third, there was a low sample size of female smoker and drinker in the studied sample, and larger sample size for these subsample will be considered for the further study. In addition, the sample included only Chinese subjects; thus, generalisability of the results to a wider population should be undertaken with caution.

## Conclusion

In conclusion, a higher serum concentration of arachidonic acid was associated with a lower change of having T2DM among Chinese men. In addition, the current results suggest sex-specific differences in the association between n-6 PUFAs and T2DM in Chinese people, and a favourable impact of arachidonic acid on T2DM, especially in Chinese men. Further research is needed to elucidate the sex effects on PUFA metabolism and the mechanisms involved.

## Data Availability Statement

The datasets used and analyzed during the current study available from the corresponding authors on reasonable request.

## Ethics Statement

The studies involving human participants were reviewed and approved by Ethics Committee of the Affiliated Zhongshan Hospital of Dalian University. The patients/participants provided their written informed consent to participate in this study.

## Author Contributions

YaL and ST: conceptualization and supervision. MB: data acquisition. YB and XC: statistical analysis. ST and YaL: investigation and writing-review and editing. YinL, HS, and YikL: writing-original draught. All authors have read and agreed to the published version of the manuscript.

## Funding

This study was supported by National Natural Science Foundation of China (81803329). The funders of the study had no role in the data collection and analysis, interpretation of this work, or the decision to submit this work for publication.

## Conflict of Interest

The authors declare that the research was conducted in the absence of any commercial or financial relationships that could be construed as a potential conflict of interest.

## Publisher's Note

All claims expressed in this article are solely those of the authors and do not necessarily represent those of their affiliated organizations, or those of the publisher, the editors and the reviewers. Any product that may be evaluated in this article, or claim that may be made by its manufacturer, is not guaranteed or endorsed by the publisher.
